# Statistical Assumptions of Substantive Analyses Across the General Linear Model: A Mini-Review

**DOI:** 10.3389/fpsyg.2012.00322

**Published:** 2012-08-28

**Authors:** Kim F. Nimon

**Affiliations:** ^1^Learning Technologies, University North TexasDenton, TX, USA

**Keywords:** assumptions, robustness, analyzing data, normality, homogeneity

## Abstract

The validity of inferences drawn from statistical test results depends on how well data meet associated assumptions. Yet, research (e.g., Hoekstra et al., 2012) indicates that such assumptions are rarely reported in literature and that some researchers might be unfamiliar with the techniques and remedies that are pertinent to the statistical tests they conduct. This article seeks to support researchers by concisely reviewing key statistical assumptions associated with substantive statistical tests across the general linear model. Additionally, the article reviews techniques to check for statistical assumptions and identifies remedies and problems if data do not meet the necessary assumptions.

The degree to which valid inferences may be drawn from the results of inferential statistics depends upon the sampling technique and the characteristics of population data. This dependency stems from the fact that statistical analyses assume that sample(s) and population(s) meet certain conditions. These conditions are called statistical assumptions. If violations of statistical assumptions are not appropriately addressed, results may be interpreted incorrectly. In particular, when statistical assumptions are violated, the probability of a test statistic may be inaccurate, distorting Type I or Type II error rates.

This article focuses on the assumptions associated with substantive statistical analyses across the general linear model (GLM), as research indicates they are reported with more frequency in educational and psychological research than analyses focusing on measurement (cf. Kieffer et al., 2001; Zientek et al., 2008). This review is organized around Table 1, which relates key statistical assumptions to associated analyses and classifies them into the following categories: randomization, independence, measurement, normality, linearity, and variance. Note that the assumptions of independence, measurement, normality, linearity, and variance apply to population data and are tested by examining sample data and using test statistics to draw inferences about the population(s) from which the sample(s) were selected.

**Table 1:** **Statistical assumptions associated with substantive analyses across the general linear model**.

Statistical analysis^a^	Independence	Measurement Level of variable(s)^b^		Normality	Linearity	Variance
		Dependent	Independent	
**CHI-SQUARE**						
Single sample	Independent observations	Nominal+	N/A			
2+ samples	Independent observations	Nominal+	Nominal+			
***T*-TEST**						
Single sample	Independent observations	Continuous	N/A	Univariate		
Dependent sample	Independent paired observations	Continuous	N/A	Univariate		
Independent sample	Independent observations	Continuous	Dichotomous	Univariate		Homogeneity of variance
**OVA-RELATED TESTS**						
ANOVA	Independent observations	Continuous	Nominal	Univariate		Homogeneity of variance
ANCOVA^c^	Independent observations	Continuous	Nominal	Univariate	✓	Homogeneity of variance
RM ANOVA	Independent repeated observations	Continuous	Nominal (opt.)	Multivariate	✓	Sphericity
MANOVA	Independent observations	Continuous	Nominal	Multivariate	✓	Homogeneity of covariance matrix
MANCOVA^c^	Independent observations	Continuous	Nominal	Multivariate	✓	Homogeneity of covariance matrix
**REGRESSION**						
Simple linear	Independent observations	Continuous	Continuous	Bivariate	✓	
Multiple linear	Independent observations	Continuous	Continuous	Multivariate	✓	Homoscedasticity
Canonical correlation	Independent observations	Continuous	Continuous	Multivariate	✓	Homoscedasticity

*^a^Across all analyses, data are assumed to be randomly sampled from the population. ^b^Data are assumed to be reliable. ^c^ANCOVA and MANCOVA also assumes homogeneity of regression and continuous covariate(s). Continuous refers to data that may be dichotomous, ordinal, interval, or ratio (cf. Tabachnick and Fidell, 2001)*.

## Randomization

A basic statistical assumption across the GLM is that sample data are drawn randomly from the population. However, much social science research is based on unrepresentative samples (Thompson, 2006) and many quantitative researchers select a sample that suits the purpose of the study and that is convenient (Gall et al., 2007). When the assumption of random sampling is not met, inferences to the population become difficult. In this case, researchers should describe the sample and population in sufficient detail to justify that the sample was at least representative of the intended population (Wilkinson and APA Task Force on Statistical Inference, 1999). If such a justification is not made, readers are left to their own interpretation as to the generalizability of the results.

## Independence

Across GLM analyses, it is assumed that observations are independent of each other. In quantitative research, data often do not meet the independence assumption. The simplest case of non-independent data is paired sample or repeated measures data. In these cases, only pairs of observations (or sets of repeated data) can be independent because the structure of the data is by design paired (or repeated). More complex data structures that do not meet the assumption of independence include nested data (e.g., employees within teams and teams within departments) and cross-classified data (e.g., students within schools and neighborhoods).

When data do not meet the assumption of independence, the accuracy of the test statistics (e.g., *t*, *F*, χ^2^) resulting from a GLM analysis depends on the test conducted. For data that is paired (e.g., pretest-posttest, parent-child), paired samples *t* test is an appropriate statistical analysis as long as the pairs of observations are independent and all other statistical assumptions (see Table 1) are met. Similarly, for repeated measures data, repeated measures ANOVA is an appropriate statistical analysis as long as sets of repeated measures data are independent and all other statistical assumptions (see Table 1) are met. For repeated measures and/or non-repeated measures data that are nested or cross-classified, multilevel modeling (MLM) is an appropriate statistical analytic strategy because it models non-independence. Statistical tests that do not model the nested or cross-classified structure of data will lead to a higher probability of rejecting the null hypotheses (i.e., Type I error) than if an appropriate statistical analysis were performed or if the data did not violate the independence assumption (Osborne, 2000).

Presuming that statistical assumptions can be met, MLM can be used to conduct statistical analyses equivalent to those listed in Table 1 (see Garson, 2012 for a guide to a variety of MLM analyses). Because multilevel models are generalizations of multiple regression models (Kreft and de Leeuw, 1998), MLM analyses have assumptions similar to analyses that do not model multilevel data. When violated, distortions in Type I and Type II error rates are imminent (Onwuegbuzie and Daniel, 2003).

## Measurement

### Reliability

Another basic assumption across the GLM is that population data are measured without error. However, in psychological and educational research, many variables are difficult to measure and yield observed data with imperfect reliabilities (Osborne and Waters, 2002). Unreliable data stems from systematic and random errors of measurement where systematic errors of measurement are “those which consistently affect an individual’s score because of some particular characteristic of the person or the test that has nothing to do with the construct being measured (Crocker and Algina, 1986, p. 105) and random errors of measurement are those which “affect an individual’s score because of purely chance happenings” (Crocker and Algina, 1986, p. 106).

Statistical analyses on unreliable data may cause effects to be underestimated which increase the risk of Type II errors (Onwuegbuzie and Daniel, 2003). Alternatively in the presence of correlated error, unreliable data may cause effects to be overestimated which increase the risk of Type I errors (Nimon et al., 2012).

To satisfy the assumption of error-free data, researchers may conduct and report analyses based on latent variables in lieu of observed variables. Such analyses are based on a technique called structural equation modeling (SEM). In SEM, latent variables are formed from item scores, the former of which become the unit of analyses (see Schumacker and Lomax, 2004 for an accessible introduction). Analyses based on latent-scale scores yield statistics as if multiple-item scale scores had been measured without error. All of the analyses in Table 1 as well as MLM analyses can be conducted with SEM. The remaining statistical assumptions apply when latent-scale scores are analyzed through SEM.

Since SEM is a large sample technique (see Kline, 2005), researchers may alternatively choose to delete one or two items in order to raise the reliability of an observed score. Although “extensive revisions to prior scale dimensionality are questionable… one or a few items may well be deleted” in order to increase reliability (Dillon and Bearden, 2001, p. 69). The process of item deletion should be reported, accompanied by estimates of the reliability of the data with and without the deleted items (Nimon et al., 2012).

### Measurement level

Table 1 denotes measurement level as a statistical assumption. Whether level of measurement is considered a statistical assumption is a point of debate in statistical literature. For example, proponents of Stevens (1946, 1951) argue that the dependent variable in parametric tests such as *t* tests and analysis-of-variance related tests should be scaled at the interval or ratio level (Maxwell and Delaney, 2004). Others (e.g., Howell, 1992; Harris, 2001) indicate that the validity of statistical conclusions depends only on whether data meet distributional assumptions not on the scaling procedures used to obtain data (Warner, 2008). Because measurement level plays a pivotal role in statistical analyses decision trees (e.g., Tabachnick and Fidell, 2001, pp. 27–29), Table 1 relates measurement level to statistical analyses from a pragmatic perspective. It is important to note that lowering the measurement level of data (e.g., dichotomizing intervally scaled data) is ill-advised unless data meet certain characteristics (e.g., multiple modes, serious skewness; Kerlinger, 1986). Although such a transformation makes data congruent with statistics that assume only the nominal measurement level, it discards important information and may produce misleading or erroneous information (see Thompson, 1988).

## Normality

For many inferential statistics reported in educational and psychological research (cf. Kieffer et al., 2001; Zientek et al., 2008), there is an assumption that population data are normally distributed. The requirement for, type of, and loci of normality assumption depend on the analysis conducted. Univariate group comparison tests (*t* tests, ANOVA, ANCOVA) assume univariate normality (Warner, 2008). Simple linear regression assumes bivariate normality (Warner, 2008). Multivariate analyses (repeated measures ANOVA, MANOVA, MANCOVA, multiple linear regression, and canonical correlation) assume multivariate normality (cf. Tabachnick and Fidell, 2001; Stevens, 2002). For analysis-of-variance type tests (OVA-type tests) involving multiple samples, the normality assumption applies to each level of the IV.

### Univariate

The assumption of univariate normality is met when a distribution of scores is symmetrical and when there is an appropriate proportion of distributional height to width (Thompson, 2006). To assess univariate normality, researchers may conduct graphical or non-graphical tests (Stevens, 2002): Non-graphical tests include the chi-square goodness of fit test, the Kolmogorov–Smirnov test, the Shapiro–Wilks test, and the evaluation of kurtosis and skewness values. Graphical tests include the normality probability plot and the histogram (or stem-and-leave plot).

Non-graphical tests are preferred for small to moderate sample sizes, with the Shapiro–Wilks test and the evaluation of kurtosis and skewness values being preferred methods for sample sizes of less than 20 (Stevens, 2002). The normal probability plot in which observations are ordered in increasing degrees of magnitude and then plotted against expected normal distribution values is preferred over histograms (or stem-and leave plots). Evaluating normality by examining the shape of histogram scan be problematic (Thompson, 2006), because there are *infinitely* different distribution shapes that may be normal (Bump, 1991). The bell-shaped distribution that many educational professionals are familiar with is not the only normal distribution (Henson, 1999).

### Bivariate

The assumption of bivariate normality is met when the linear relationship between two variables follows a normal distribution (Burdenski, 2000). A necessary but insufficient condition for bivariate normality is univariate normality for each variable. Bivariate normality can be evaluated graphically (e.g., scatterplots). However, in practice, even large datasets (*n* > 200) have insufficient data points to evaluate bivariate normality (Warner, 2008) which may explain why this assumption often goes unchecked and unreported.

### Multivariate

The assumption of multivariate normality is met when each variable in a set is normally distributed around fixed values on all other variables in the set (Henson, 1999). Necessary but insufficient conditions for multivariate normality include univariate normality for each variable along with bivariate normality for each variable pair. Multivariate normality can be assessed graphically or with statistical tests.

To assess multivariate normality graphically, a scatterplot of Mahalanobis distances and paired χ^2^-values may be examined, where Mahalanobis distance indicates how far each “set of scores is from the group means adjusting for correlation of the variables” (Burdenski, 2000, p. 20). If the plot approximates a straight-line, data are considered multivariate normal. Software to produce the Mahalanobis distance by χ^2^ scatterplot can be found in Thompson (1990); Henson (1999), and Fan (1996).

Researchers may also assess multivariate normality by testing Mardia’s (1985) coefficient of multivariate kurtosis and examining its critical ratio. If the critical ratio of Mardia’s coefficient of multivariate kurtosis is less than 1.96, a sample can be considered multivariate normal at the 0.05 significance level, indicating that the multivariate kurtosis is not statistically significantly different than zero. Mardia’s coefficient of multivariate kurtosis is available in statistical software packages including AMOS, EQS, LISREL, and PASW (see DeCarlo, 1997).

### Violations

The effect of violating the assumption of normality depends on the level of non-normality and the statistical test examined. As long the assumption of normality is not severely violated, the actual Type I error rates approximate nominal rates for *t* tests and OVA-tests (cf. Boneau, 1960; Glass et al., 1972; Stevens, 2002). However, in the case of data that are severely platykurtic, power is reduced in *t* tests and OVA-type tests (cf. Boneau, 1960; Glass et al., 1972; Stevens, 2002). Non-normal variables that are highly skewed or kurtotic distort relationships and significance tests in linear regression (Osborne and Waters, 2002). Similarly, proper inferences regarding statistical significance tests in canonical correlation depend on multivariate normality (Tabachnick and Fidell, 2001). If the normality assumption is violated, researchers may delete outlying cases, transform data, or conduct non-parametric tests (see Conover, 1999; Osborne, 2012), as long as the process is clearly reported.

## Linearity

For parametric statistics involving two or more continuous variables (ANCOVA, repeated measures ANOVA, MANOVA, MANCOVA, linear regression, and canonical correlation) linearity between pairs of continuous variables is assumed (cf. Tabachnick and Fidell, 2001; Warner, 2008). The assumption of linearity is that there is a straight-line relationship between two variables. Linearity is important in a practical sense because Pearson’s *r*, which is fundamental to the vast majority of parametric statistical procedures (Graham, 2008), captures *only* the linear relationship among variables (Tabachnick and Fidell, 2001). Pearson’s *r* underestimates the true relationship between two variables that is non-linear (i.e., curvilinear; Warner, 2008).

Unless there is strong theory specifying non-linear relationships, researchers may assume linear relationships in their data (Cohen et al., 2003). However, linearity is not guaranteed and should be validated with graphical methods (see Tabachnick and Fidell, 2001). Non-linearity reduces the power of statistical tests such as ANCOVA, MANOVA, MANCOVA, linear regression, and canonical correlation (Tabachnick and Fidell, 2001). In the case of ANCOVA and MANCOVA, non-linearity results in improper adjusted means (Stevens, 2002). If non-linearity is detected, researchers may transform data, incorporate curvilinear components, eliminate the variable producing non-linearity, or conduct a non-linear analysis (cf. Tabachnick and Fidell, 2001; Osborne and Waters, 2002; Stevens, 2002; Osborne, 2012), as long as the process is clearly reported.

## Variance

Across parametric statistical procedures commonly used in quantitative research, at least five assumptions relate to variance. These are: homogeneity of variance, homogeneity of regression, sphericity, homoscedasticity, and homogeneity of variance-covariance matrix.

Homogeneity of variance applies to univariate group analyses (independent samples *t* test, ANOVA, ANCOVA) and assumes that the variance of the DV is roughly the same at all levels of the IV (Warner, 2008). The Levene’s test validates this assumption, where smaller statistics indicate greater homogeneity. Research (Boneau, 1960; Glass et al., 1972) indicates that univariate group analyses are generally robust to moderate violations of homogeneity of variance as long as the sample sizes in each group are approximately equal. However, with unequal sample sizes, heterogeneity may compromise the validity of null hypothesis decisions. Large sample variances from small-group sizes increase the risk of Type I error. Large sample variances from large-group sizes increase the risk of Type II error. When the assumption of homogeneity of variance is violated, researchers may conduct and report non-parametric tests such as the Kruskal–Wallis. However, Maxwell and Delaney (2004) noted that the Kruskal–Wallis test also assumes equal variances and suggested that data be either transformed to meet the assumption of homogeneity of variance or analyzed with tests such as Brown–Forsythe F^*^ or Welch’s W.

Homogeneity of regression applies to group analyses with covariates, including ANCOVA and MANCOVA, and assumes that the regression between covariate(s) and DV(s) in one group is the same as the regression in other groups (Tabachnick and Fidell, 2001). This assumption can be examined graphically or by conducting a statistical test on the interaction between the COV(s) and the IV(s). Violation of this assumption can lead to very misleading results if covariance is used (Stevens, 2002). For example, in the case of heterogeneous slopes, group means that have been adjusted by a covariate could indicate no difference when, in fact, group differences might exist at different values of the covariate. If heterogeneity of regression exists, ANCOVA and MANCOVA are inappropriate analytic strategies (Tabachnick and Fidell, 2001).

Sphericity applies to repeated measures analyses that involve three or more measurement occasions (repeated measures ANOVA) and assumes that the variances of the differences for all pairs of repeated measures are equal (Stevens, 2002). Presuming that data are multivariate normal, the Mauchly test can be used to test this assumption, where smaller statistics indicate greater levels of sphericity (Tabachnick and Fidell, 2001). Violating the sphericity assumption increases the risk of Type I error (Box, 1954). To adjust for this risk and provide better control for Type I error rate, the degrees of freedom for the repeated measures *F* test may be corrected using and reporting one of three adjustments: (a) Greenhouse–Geisser, (b) Huynh–Feldt, and (c) Lower-bound (see Nimon and Williams, 2009). Alternatively, researchers may conduct and report analyses that do not assume sphericity (e.g., MANOVA).

Homoscedasticity applies to multiple linear regression and canonical correlation and assumes that the variability in scores for one continuous variable is roughly the same at all values of another continuous variable (Tabachnick and Fidell, 2001). Scatterplots are typically used to test homoscedasticity. Linear regression is generally robust to slight violations of homoscedasticity; however, marked heteroscedasticity increases the risk of Type I error (Osborne and Waters, 2002). Canonical correlation performs best when relationships among pairs of variables are homoscedastic (Tabachnick and Fidell, 2001). If the homoscedasticity assumption is violated, researchers may delete outlying cases, transform data, or conduct non-parametric tests (see Conover, 1999; Osborne, 2012), as long as the process is clearly reported.

Homogeneity of variance-covariance matrix is a multivariate generalization of homogeneity of variance. It applies to multivariate group analyses (MANOVA and MANCOVA) and assumes that the variance-covariance matrix is roughly the same at all levels of the IV (Stevens, 2002). The Box M test tests this assumption, where smaller statistics indicate greater homogeneity. Tabachnick and Fidell (2001) provided the following guidelines for interpreting violations of this assumption: if sample sizes are equal, heterogeneity is not an issue. However, with unequal sample sizes, heterogeneity may compromise the validity of null hypothesis decisions. Large sample variances from small-group sizes increase the risk of Type I error whereas large sample variances from large-group sizes increase the risk of Type II error. If sample sizes are unequal and the Box M test is significant at *p* < 0.001, researchers should conduct the Pillai’s test or equalize sample sizes by random deletion of cases if power can be retained.

## Discussion

With the advances in statistical software, it is easy for researchers to use point and click methods to conduct a wide variety of statistical analyses on their datasets. However, the output from statistical software packages typically does not fully indicate if necessary statistical assumptions have been met. I invite editors and reviewers to use the information presented in this article as a basic checklist of the statistical assumptions to be reported in scholarly reports. The references cited in this article should also be helpful to researchers who are unfamiliar with a particular assumption or how to test it. For example, Osborne’s (2012) book provides an accessible treatment of a wide variety of data transformation techniques while Burdenski’s (2000) article review graphics procedures to evaluate univariate, bivariate, and multivariate normality. Finally, the information presented in this article should be helpful to readers of scholarly reports. Readers cannot presume that just because an article has survived peer review, the interpretation of the findings is methodologically sound (cf. Henson et al., 2010). Readers must make their own judgment as to the quality of the study if information that could affect the validity of the data presented is not reported. With the information presented in this article and others, I invite readers to take an active role in evaluating the findings of quantitative research reports and become informed consumers of the data presented.

## Conflict of Interest Statement

The author declares that the research was conducted in the absence of any commercial or financial relationships that could be construed as a potential conflict of interest.

## References

[B1] Boneau C. A. (1960). The effects of violations of assumptions underlying the t test. Psychol. Bull. 57, 49–64 10.1037/h0041412 13802482

[B2] Box G. E. P. (1954). Some theorems on quadratic forms applied in the study of analysis of variance problems, II. Effects of inequality of variance and of correlation between errors in the two-way classification. Ann. Math. Statist. 25, 484–498 10.1214/aoms/1177728786

[B3] Bump W. (1991). The normal curve takes many forms: a review of skewness and kurtosis. Paper Presented at the Annual Meeting of the Southwest Educational Research Association, San Antonio [ERIC Document Reproduction Service No. ED 342 790].

[B4] Burdenski T. K. (2000). Evaluating univariate, bivariate, and multivariate normality using graphical procedures. Mult. Linear Regression Viewp. 26, 15–28

[B5] Cohen J. Cohen P. West S. G. Aiken L. S. (2003). Applied Multiple Regression/Correlation Analysis for the Behavioral Sciences, 3rd Edn. Mahwah, NJ: Erlbaum

[B6] Conover W. J. (1999). Practical Nonparametric Statistics, 3rd Edn. New York: Wiley

[B7] Crocker L. Algina J. (1986). Introduction to Classical and Modern Test Theory. Belmont, CA: Wadsworth

[B8] DeCarlo L. T. (1997). On the meaning and use of kurtosis. Psychol. Methods 2, 292–307 10.1037/1082-989X.2.3.292

[B9] Dillon W. R. Bearden W. (2001). Writing the survey question to capture the concept. J. Consum. Psychol. 10, 67–69

[B10] Fan X. (1996). A SAS program for assessing multivariate normality. Educ. Psychol. Meas. 56, 668–674 10.1177/0013164496056003001

[B11] Gall M. D. Gall J. P. Borg W. R. (2007). Educational Research: An Introduction, 8th Edn. Boston, MA: Allyn and Bacon

[B12] Garson G. D. (2012). Hierarchical Linear Modeling: Guide and Applications. Thousand Oaks, CA: Sage Publications, Inc.

[B13] Glass G. V. Peckham P. D. Sanders J. R. (1972). Consequences of failure to meet assumptions underlying the fixed effects analyses of variance and covariance. Rev. Educ. Res. 42, 237–288 10.3102/00346543042003237

[B14] Graham J. M. (2008). The general linear model as structural equation modeling. J. Educ. Behav. Stat. 33, 485–506 10.3102/1076998607306151

[B15] Harris R. J. (2001). A Primer of Multivariate Statistics, 3rd Edn. Mahwah, NJ: Erlbaum

[B16] Henson R. K. (1999). “Multivariate normality: what is it and how is it assessed?” in Advances in Social Science Methodology, Vol. 5, ed. Thompson B. (Stamford, CT: JAI Press), 193–211

[B17] Henson R. K. Hull D. M. Williams C. S. (2010). Methodology in our education research culture: toward a stronger collective quantitative proficiency. Educ. Res. 39, 229–240 10.3102/0013189X10365102

[B18] Hoekstra R. Kiers H. A. L. Johnson A. (2012). Are assumptions of well-known statistical techniques checked, and why (not)? Front. Psychol. 3:137 10.3389/fpsyg.2012.00137 22593746 PMC3350940

[B19] Howell D. D. (1992). Statistical Methods for Psychology, 3rd Edn. Boston: PWS-Kent

[B20] Kerlinger F. N. (1986). Foundations of Behavioral Research, 3rd Edn. New York: Holt, Rinehart and Winston

[B21] Kieffer K. M. Reese R. J. Thompson B. (2001). Statistical techniques employed in AERJ and JCP articles from 1988 to 1997: a methodological review. J. Exp. Educ. 69, 280–309 10.1080/00220970109599489

[B22] Kline R. B. (2005). Principles and Practice of Structural Equation Modeling, 2nd Edn. New York, NY: The Guilford Press

[B23] Kreft I. G. G. de Leeuw J. (1998). Introducing Multilevel Modeling. Thousand Oaks, CA: Sage

[B24] Mardia K. V. (1985). “Mardia’s test of multinormality,” in Encyclopedia of Statistical Sciences, Vol. 5, eds Kotz S. Johnson N. L. (New York: Wiley), 217–221

[B25] Maxwell S. E. Delaney H. D. (2004). Designing Experiments and Analyzing Data: A Model Comparison Perspective, 2nd Edn. Mahwah, NJ: Erlbaum

[B26] Nimon K. Williams C. (2009). Performance improvement through repeated measures: a primer for educators considering univariate and multivariate design. Res. High. Educ. J. 2, 117–136

[B27] Nimon K. Zientek L. R. Henson R. (2012). The assumption of a reliable instrument and other pitfalls to avoid when considering the reliability of data. Front. Psychol. 3:102 10.3389/fpsyg.2012.00102 22518107 PMC3324779

[B28] Onwuegbuzie A. J. Daniel L. G. (2003). “Typology of analytical and interpretational errors in quantitative and qualitative educational research,” in Current Issues in Education, Vol. 6 Available at: http://cie.ed.asu.edu/volume6/number2/ [accessed February 19, 2003].

[B29] Osborne J. Waters E. (2002). Four assumptions of multiple regression that researchers should always test. Pract. Assess. Res. Eval. 8 Available at: http://PAREonline.net/getvn.asp?v=8&n=2

[B30] Osborne J. W. (2000). Advantages of hierarchical linear modeling. Pract. Assess. Res. Eval. 71 Available at: http://pareonline.net/getvn.asp?v=7&n=1

[B31] Osborne J. W. (2012). Best Practices in Data Cleaning: A Complete Guide to Everything You Need to Do Before and After Collecting Your Data. Thousand Oaks, CA: Sage

[B32] Schumacker R. E. Lomax R. G. (2004). A Beginner’s Guide to Structural Equation Modeling. Mahwah, NJ: Erlbaum

[B33] Stevens J. (2002). Applied Multivariate Statistics for the Social Sciences, 4th Edn. Mahwah, NJ: Erlbaum

[B34] Stevens S. (1946). On the theory of scales of measurement. Science 103, 677–680 10.1126/science.103.2684.677 17750512

[B35] Stevens S. (1951). “Mathematics, measurement, and psychophysics,” in Handbook of Experimental Psychology, ed. Stevens S. (New York: Wiley), 1–49

[B36] Tabachnick B. G. Fidell L. S. (2001). Using Multivariate Statistics, 4th Edn. Needham Heights, MA: Allyn and Bacon

[B37] Thompson B. (1988). Discard variance: a cardinal since in research. Meas. Eval. Couns. Dev. 21, 3–4

[B38] Thompson B. (1990). Multinor: a Fortran program that assists in evaluating multivariate normality. Educ. Psychol. Meas. 50, 845–848 10.1177/0013164490502011

[B39] Thompson B. (2006). Foundations of Behavioral Statistics: An Insight-Based Approach. New York: Guilford Press

[B40] Warner R. M. (2008). Applied Statistics: From Bivariate through Multivariate Techniques. Thousand Oaks, CA: Sage

[B41] Wilkinson L. APA Task Force on Statistical Inference (1999). Statistical methods in psychology journals: guidelines and explanation. Am. Psychol. 54, 594–604 10.1037/0003-066X.54.8.594

[B42] Zientek L. R. Capraro M. M. Capraro R. M. (2008). Reporting practices in quantitative teacher education research: one look at the evidence cited in the AERA panel report. Educ. Res. 37, 208–216 10.3102/0013189X08319762

